# Psychometric evaluation of the depression anxiety stress scale 8-items (DASS-8)/DASS-12/DASS-21 among family caregivers of patients with dementia

**DOI:** 10.3389/fpubh.2022.1012311

**Published:** 2022-10-25

**Authors:** Amira Mohammed Ali, Rana Ali Alameri, Amin Omar Hendawy, Rasmieh Al-Amer, Ghada Shahrour, Esraa M. Ali, Abdulmajeed A. Alkhamees, Nashwa Ibrahim, Bothaina Hussein Hassan

**Affiliations:** ^1^Department of Psychiatric Nursing and Mental Health, Faculty of Nursing, Alexandria University, Alexandria, Egypt; ^2^Fundamentals of Nursing Department, College of Nursing, Imam Abdulrahman Bin Faisal University, Dammam, Saudi Arabia; ^3^Department of Animal and Poultry Production, Faculty of Agriculture, Damanhour University, Damanhour, Egypt; ^4^Faculty of Nursing, Isra University, Amman, Jordan; ^5^School of Nursing and Midwifery, Western Sydney University, Penrith, NSW, Australia; ^6^Jordan University of Science and Technology, Faculty of Nursing, Irbid, Jordan; ^7^Department of Basic and Educational Sciences, Faculty of Education for Early Childhood, Alexandria University, Alexandria, Egypt; ^8^Department of Medicine, Unayzah College of Medicine and Medical Sciences, Qassim University, Unayzah, Saudi Arabia; ^9^Psychiatric and Mental Health Nursing Department, Faculty of Nursing, Mansoura University, Mansoura, Egypt; ^10^Department of Nursing, College of Applied Medical Sciences, King Faisal University, Al-Ahsa, Saudi Arabia; ^11^Department of Gerontological Nursing, Faculty of Nursing, Alexandria University, Alexandria, Egypt

**Keywords:** psychological distress/anxiety/depression, dementia/cognitive impairment/Alzheimer's disease/Parkinson's disease, short form of the Depression Anxiety Stress Scale 21/Depression Anxiety Stress Scale 8-items, factor structure/psychometric properties/structural validity/criterion validity/known-group validity/validation/measurement invariance/discriminant validity, old age/elders/elderly, loneliness, informal/family caregivers, spouse/adult children

## Abstract

Patients with dementia express a set of problematic and deteriorating symptoms, along with self-care dependency. Over time, the mental health of family caregivers of persons with dementia may be affected, putting them at a high risk for psychopathology, which may be associated with endangered wellbeing of people with dementia. This cross-sectional instrumental design study examined the psychometric properties of the Depression Anxiety Stress Scale 8-items (DASS-8), DASS-12, and DASS-21 in a convenient sample of 571 caregivers from northern Italy and southern Switzerland (mean age = 53 years, SD = 12, range = 24–89 years). A bifactor structure of the three measures had the best fit; some items of the DASS-12/DASS-21 failed to load on their domain-specific factors. The three-factor structure was invariant across various groups (e.g., gender and education), expressed adequate reliability and convergent validity, and had strong positive correlation with the three-item UCLA Loneliness Scale (UCLALS3). Distress scores did not differ among carers of different types of dementia (Alzheimer's disease vs. other types, e.g., vascular dementia). However, distress scores were significantly high among female individuals, adult children caregivers, those caring for dependent patients, and those who received help with care. For 54.9 and 38.8% of the latter, care was provided by relatives and health professionals, respectively. Since the DASS-8 expresses adequate psychometrics comparable with the DASS-21, it may be used as a brief measure of distress in this population.

## Introduction

Dementia is a devastating clinical syndrome, which represents the second prevalent neurological condition after headache and the third most burdensome disease—striking more than 50 million people worldwide and contributing to an annual cost of care of more than $232 billion in the United States (1–4). The most common form of dementia is Alzheimer's disease (60–80% of dementia cases) (1, 5). However, it may develop in Parkinson's disease, cerebral vascular injury, metabolic disorders, etc. (1, 3, 6, 7). Dementia runs a progressive course. Drastic deteriorations in cognitive and functional performance develop during late stages of the disease (2, 3). Thus, dementia represents a major source of disability, with most patients expressing significant impairments in all aspects of life and high dependency in all activities of daily living (ADL). Dementia care is largely provided by family members, friends, or informal caregivers (2, 3, 8).

More than two-thirds of family caregivers of patients with dementia in the United States perform numerous medical/nursing tasks, which are usually performed by health professionals such as managing multiple medications, injections, tube feedings, and wound care (9). Family caregivers are stressed with dementia symptoms (e.g., cognitive alterations, anxiety, agitation, disinhibition, aggressive behavior, and sleep disturbances), comorbidities, and complex medication regimen (8, 9). Moreover, family caregivers are primarily elderly spouses (mean age = 62.5 ± 23.3 years, 74.1% women), who may endure physical and mental adversities associated with their own old age (e.g., age-related diseases and disability) (9, 10). As a result, caregivers frequently experience burnout, emotional distress, anxiety, sleep disturbance, poor general health, low quality of life, and social isolation (8, 9, 11–13), with higher vulnerability among women, spouses, and elders, especially those with deficient coping, social isolation, lack of training/information about the disease, poor premorbid relationship with care recipients, and high levels of negative expressed emotions (8, 9). Caregiving distress among adult-child caregivers of parents with dementia predominately originates from the impact of caregiving on children's health, schedule, and finance (14).

Orchestrated with the overall rise in distress among the general population during the COVID-19 pandemic (15), caregivers of patients with dementia have exhibited a range of mental symptoms such as mood dysfunction (e.g., anxiety and depression), sleep disturbance, loneliness, and dysfunctional eating (16, 17). Increased caregiver distress is reported to be a direct effect of COVID-19 confinement, independent of dementia stage. It is also associated with family caregivers' concerns about unavailability of paid caregivers and fear of transmitting COVID-19 infection while caring for their relatives (18). In addition, the COVID-19 era has witnessed an increase in the severity of dementia symptoms: behavioral dysfunctions, anxiety, apathy/depression, and an excessive decline in cognitive functions (18, 19). Deteriorations in dementia symptoms during COVID-19 are associated with increased caregiver distress, as well as increased intensity of caregiving and severity of caregiver burden (16, 18, 20). Distress among family and informal caregivers can adversely affect the dementia course, leading to further deteriorations in the cognitive, behavioral, and emotional symptoms of dementia, in addition to the institutionalization of dementia care recipients and increased elder abuse (8, 10). Therefore, proper assessment of distress symptomatology among dementia caregivers is necessary to mobilize actions, which are necessary to facilitate resilience in such a vulnerable group.

According to the tripartite model, general affective distress is a common component of both depression and anxiety. However, both conditions are suggested to have distinct features, which can be reliably measured (21). The Depression Anxiety Stress Scale-21 (DASS-21) has been designed and is commonly used to measure the distinct features of depression, anxiety, and stress (22). Nonetheless, subsequent investigations revealed failure of the DASS-21 to express a consistent dimensional structure (23–27), along with concerns about its psychometric equivalence across different groups both in English-speaking countries and other parts of the world (23, 28–30), as well as a ceiling effect (31). Accordingly, the scale has undergone extensive revisions, resulting in several brief forms with better psychometric properties [DASS-18 (32, 33), DASS-14 (34), DASS-13, DASS-9 (23), DASS-12 (35), and DASS-8 (36)]. Given that short scales encourage higher response rates, the last two shortened versions of the DASS-21 have been recently tested among psychiatric patients from Korea and Saudi Arabia; healthy individuals from the USA, Australia, Saudi Arabia, and Ghana; and Australian women with chronic pelvic pain (35–38). In all studies, the DASS-8 expressed the best fit and invariance across different groups. Its internal consistency and convergent validity were close to or greater than those of the parent scale and the DASS-12. Discriminant validity analysis revealed that the subscales of the DASS-8 are more distinct than those of the parent scale and the DASS-12 (37, 38). Because of its brevity and simplicity, the DASS-8 seems to be a more attractive measure of general distress and mental symptoms of depression, anxiety, and stress. However, individuals from different cultures have their own unique ways of responding to stressful events and reporting their mental distress. Such variations may affect the manner through which they respond to the items of a symptom scale, resulting in a reporting bias, which may reduce the credibility of measurement (39). Therefore, further investigations of the psychometric characteristics of the DASS-8 in various cultural contexts and among different groups are needed, should the scale be used as a global measure of common mental symptomatology. This study aimed to evaluate the psychometric properties of the DASS-8 relative to the DAS-12 and the DASS-21 among dementia family caregivers from Italy and Switzerland. Based on previous studies, we hypothesized that the DASS measures will express a consistent three-dimensional structure and measurement invariance among caregivers from both countries. The DASS measures would congruently have positive correlations with caregiver loneliness. Based on the literature (8–10, 14), distress levels are expected to be higher among respondents who are females, spouses of care recipients, those not receiving help with care, and those caring for patients with Alzheimer's disease or who are ADL-dependent patients than in those who are males, adult children, receiving help, caring for other types of dementia, or autonomous patients.

## Materials and methods

### Study design, participants, and procedure

This cross-sectional study is a secondary analysis of a public dataset (40) comprising a convenient sample of Italian-speaking adult family caregivers of people with dementia. Participants were recruited through advertisements disseminated through social media and 53 dementia day-care centers in Italy and southern Switzerland. Data were collected through an online survey implemented in Research Electronic Data Capture (RedCap) during the period between 25 May and 25 June 2020. All the participants signed a digital informed consent. The data collection procedure was approved by Italian and Swiss Cantonal ethics committees (16), and the dataset is shared under the terms of creative common license (CC BY 4.0) (40). Therefore, no ethical approval was obtained for the current study.

### Measures

The participants completed a self-administered questionnaire, which was in Italian and consisted of three sections. The first section inquired about participants' sociodemographic characteristics (age, gender, education, and employment), the type of dementia, level of ADL dependency, duration of dementia care provision, their relationship with the care recipient, and if they received help with dementia care (16).

Section two comprised the Italian version of the Depression Anxiety Stress Scale (DASS-21) (41) as a measure of psychological distress, depression, anxiety, and stress symptoms. The DASS-21 is composed of three subscales, and each subscale comprised seven items. The respondents would rate the intensity of their symptoms during the last week on a four-point scale, which ranged from 0 (did not apply to me at all) to 3 (applied to me very much or most of the time). The minimum and maximum total scores of the DASS-21 ranged between 0 and 63 (27, 33). In this instrumental design study, the short versions of the DASS-21 were nested within the parent scale, i.e., the data on the items of the short scales were obtained from the DASS-21 and analyzed as shown below. The DASS-8 is the shortest version of the DASS-21. It is composed of three subscales: depression (three items, e.g., felt that I had nothing to look forward), anxiety (three items, e.g., felt close to panic), and stress (two items, e.g., was using a lot of my mental energy) (36, 38). The minimum score of the DASS-8 and its subscales is 0, while the maximum scores are 24, 9, 9, and 6, respectively. The DASS-12 consists of three subscales; each subscale consists of four items. The minimum and maximum scores of the DASS-12 ranges from 0 to 36, while and the minimum and maximum scores of each of its three subscales range from 0 to 12 (35). For all the DASS measures, higher scores denote higher endorsement of mental distress symptoms. The reliability of the DASS-21, DASS-8, and DASS-12 in this sample is excellent (please see the Results section for the details).

Section three comprised the Italian version of the University of California, Los Angeles, Loneliness Scale-version 3 (UCLALS3) (42); three items of the UCLALS3 were used [lack of companionship, feel left out (exclusion), and feel isolated (isolation)], which represent three interrelated dimensions of isolation, relational connectedness, and trait loneliness. The frequency of endorsing items since the start of the COVID-19 outbreak is rated on a three-point Likert scale, which ranges from 1 (hardly never) to 3 (often). Thus, the minimum and maximum total scores of the current version of the UCLALS3 range between 3 and 9. Higher scores reflect higher loneliness (16, 42, 43). The reliability of the UCLALS3 in this study is very good (coefficient alpha = 0.87).

### Statistical analysis

Shapiro–Wilk W test was used to examine the distribution of different versions of the DASS and the UCLALS3. Variables with a non-normal distribution were described by median (MD) and interquartile range (IQR; Q1–Q3). Variables with a normal distribution were described by mean and standard deviation. Categorical variables were described by frequencies and percentages.

Based on the findings of previous studies (36–38), the factor structures of the DASS-8 and DASS-12 were examined using confirmatory factor analysis (CFA). In this study, four models were tested: a unidimensional structure, a three-factor structure, a second-order factor structure, and a bifactor structure. The criteria used to evaluate model fit were chi-square (χ^2^) index, comparative fit index (CFI), Tucker–Lewis Index (TLI), standardized root-mean-square residual (SRMR), and root-mean-square error of approximation (RMSEA). Ideally, χ^2^ should be non-significant. However, χ^2^ values can be greatly affected by sample size. Therefore, model fit can be parsimoniously considered good or acceptable based on the values of absolute fit measures: CFI and TLI equal to or above 0.95 and 0.90, along with SRMR and RMSEA < 0.06 and 0.08, respectively (44, 45). Based on suggestions pointed out by modification indices, few error terms were correlated to improve the model fit.

Measurement invariance of the DASS-8/DASS-12/DASS-21 was examined at the configural, metric, scalar, and strict levels (46, 47) across groups of gender, education (compulsory, high school, and university), employment (employed and non-employed), country of residence, type of dementia (Alzheimer's disease vs. all other types), level of dependency (autonomous vs. dependent), receiving help with caregiving (yes vs. no), and relationship with care recipients (spouses vs. adult children). Models with a significant χ^2^-value were considered non-invariant if ΔCFI and ΔRMSEA exceeded 0.02 and 0.015, respectively (15, 46).

To examine the known-group validity of the DASS-8/DASS-12/DASS-21, Mann–Whitney *U*-test was used to determine whether these measures and their subscales can differentiate respondents with higher distress across groups of gender, dementia type, level of dependency, and help with caregiving. To examine the discriminant validity of the DASS measures, we computed heterotrait-to-monotrait (HTMT) ratio of correlations of items comprising the DASS-8/DASS-12/DASS-21 (38, 48).

The internal consistency of the three scales and their subscales was evaluated by coefficient alpha, alpha if item deleted, and item–total correlations. The latter was also used as an indicator of convergent validity. Spearman's correlations of the DASS-8, DASS-12, and their subscales with the DASS-21 scale and its subscales were used to examine their convergent validity. The criterion validity of the DASS measures was tested by correlating their scores with the UCLALS3. Respondents with higher loneliness scores were expected to display higher levels of distress. The analyses were conducted in Amos version 24 and SPSS version 28. Significance was considered at a probability less than 0.05 in two-tailed tests.

## Results

### Characteristics of the participants

The sociodemographic characteristics of the participants (*N* = 571, mean age = 53 ± 12 years, range = 24–89 years, 74.4% Italian, and 25.6% Swiss) are described in detail elsewhere (16). In brief, most of the participants were females (81.6%) and adult children of patients with dementia (71.8%). They mostly had high school education (56.4%), were employed (49.6%), provided dementia care for an average of 6.1 (SD = 4.0) years, and received help with care from other family members, friends, or health professionals (58.7%). Alzheimer's disease was the most prevalent type of dementia (55.3%), and 79.7% of patients with dementia were dependent in activities of ADL.

### Results of confirmatory factor analysis and invariance analysis

**Figure 1:**
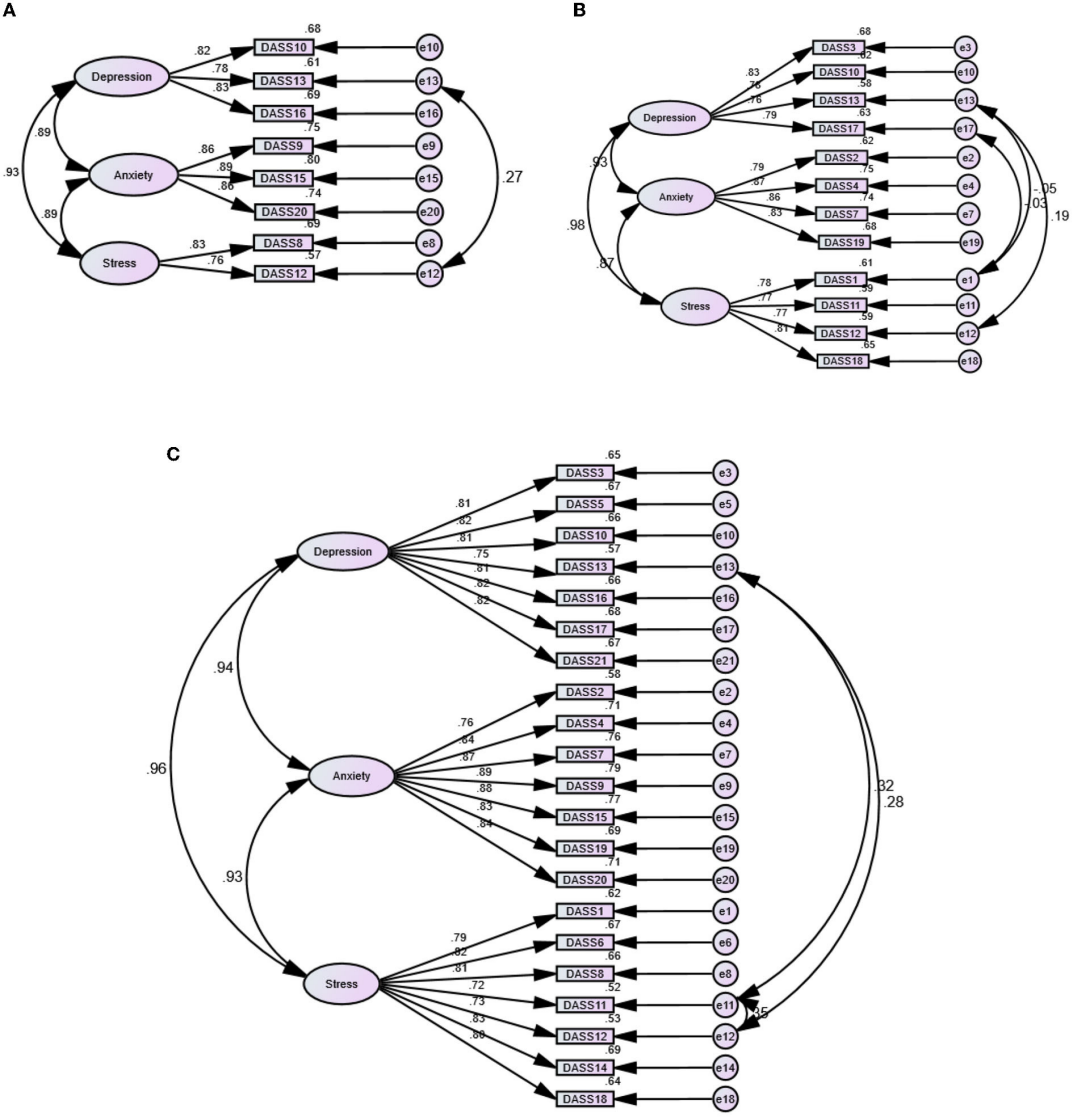
Factor structure of the Depression Anxiety Stress Scale (DASS)-21 **(C)** and its short versions: the DASS-8 **(A)** and the DASS-12 **(B)** among dementia family caregivers.

Table 1 shows poor fit of the one-factor structure of the three DASS measures. The three-factor structure of the DASS-8 and the DASS-21 had good and acceptable fit, respectively. Meanwhile, RMSEA indicated misfit of the three-factor structure of the DASS-12, even when the error terms of three items were correlated. Notably, the bifactor structures of the three scales expressed the best fit among all models. In that model, all the items of the DASS-8 loaded significantly on their domain-specific factors, albeit the loadings of items 12 and 13 on the corresponding factors were below 0.3. Simultaneously, item 13 had loadings below 0.1, while items 11 and 12 failed to load on their corresponding factors in models representing the DASS-12 and the DASS-21, respectively (Supplementary materials). Given the good fit of the three-factor structure, with considerably satisfactory item loadings (Figure 1), this model was used for testing measurement invariance of the DASS scales.

**Table 1:** Goodness of fit of the confirmatory factor analysis models representing the Depression Anxiety Stress Scale-8 (DASS-8), DASS-12, and DASS-21 among dementia family caregivers.

**Models**	**Samples**	**χ^2^**	***P***	***Df***	**CFI**	**TLI**	**RMSEA**	**RMSEA 90% CI**	**SRMR**
Model 1	Crude	212.534	0.001	20	0.942	0.919	0.130	0.114–0.146	0.0391
1F DASS-8	Correlated error	115.331	0.001	17	0.971	0.952	0.101	0.084–0.119	0.0288
Model 2	Crude	89.717	0.001	17	0.978	0.964	0.087	0.069–0.105	0.0241
3F DASS-8	Correlated error	60.321	0.012	16	0.987	0.977	0.070	0.052–0.089	0.0178
Model 3 Bifactor DASS-8	Crude	50.737	0.001	16	0.990	0.982	0.062	0.043–0.081	0.0162
Model 4	Crude	515.206	0.001	54	0.912	0.892	0.122	0.113–0.132	0.0508
1F DASS-12	Correlated error	303.428	0.001	49	0.951	0.935	0.095	0.085–0.106	0.0390
Model 5	Crude	356.390	0.001	51	0.942	0.924	0.102	0.093–0.113	0.0450
3F DASS-12	Correlated error	336.485	0.001	46	0.945	0.924	0.103	0.092–0.113	0.0429
Model 6 Bifactor DASS-12	Crude	153.312	0.001	50	0.980	0.974	0.060	0.049–0.071	0.0253
Model 7	Crude	1,279.948	0.001	189	0.903	0.892	0.101	0.095–0.106	0.0444
1F DASS-21	Correlated error	1,070.892	0.001	185	0.921	0.910	0.092	0.89–0.97	0.0406
Model 8	Crude	997.013	0.001	186	0.928	0.918	0.087	0.082–0.093	0.0404
3F DASS-21	Correlated error	864.902	0.001	183	0.939	0.930	0.081	0.075–0.086	0.0366
Model 9 Bifactor DASS-21	Crude	701.337	0.001	184	0.954	0.947	0.070	0.065–0.076	0.0328

χ^2^, chi-square; df, degrees of freedom; CFI, comparative fit index; TLI, Tucker–Lewis index; RMSEA, root mean square error of approximation; CI, confidence interval; SRMR, standardized root mean residual.

As indicated in Table 2, the three-factor structures of the DASS-8, DASS-12, and DASS-21 were invariant at the configural, metric, scalar, and strict levels across all groups. Nevertheless, the DASS-8 was non-invariant at the scalar level across country groups (ΔCFI > 0.02 and ΔRMSEA >0.15). The DASS-12 also tended to be non-invariant at the scalar level (ΔCFI > 0.02).

**Table 2:** Invariance of the three-factor structures of the Depression Anxiety Stress Scale 8 (DASS-8), DASS-12, and DASS-21 across different characteristics of dementia family caregivers.

**Model**	**Groups**	**Invariance levels**	**χ^2^**	**Df**	***P***	**Δχ^2^**	**Δdf**	***p*(Δχ^2^)**	**CFI**	**ΔCFI**	**TLI**	**ΔTLI**	**RMSEA**	**ΔRMSEA**	**SRMR**
DASS-8	Gender	Configural	74.110	32	0.001				0.987		0.977		0.048		0.0319
		Metric	78.903	37	0.001	4.792	5	0.442	0.987	0.000	0.981	−0.004	0.045	0.003	0.0342
		Strong	102.237	43	0.001	23.334	6	0.001	0.982	0.005	0.976	0.005	0.049	−0.004	0.0725
		Strict	120.553	52	0.001	18.316	9	0.032	0.979	0.003	0.977	−0.001	0.048	0.001	0.0880
DASS-12		Configural	398.202	96	0.001				0.941		0.919		0.074		0.05560
		Metric	410.241	105	0.001	12.039	9	0.211	0.940	0.001	0.925	−0.006	0.072	0.002	0.0596
		Strong	427.588	111	0.001	17.347	6	0.008	0.938	0.002	0.926	−0.001	0.071	0.001	0.0781
		Strict	451.821	126	0.001	24.233	15	0.061	0.936	0.002	0.933	−0.007	0.067	0.004	0.0867
DASS-21		Configural	1,146.243	366	0.001				0.929		0.919		0.061		0.0541
		Metric	1,163.750	384	0.001	17.507	18	0.489	0.929	0.000	0.923	−0.004	0.060	0.001	0.0586
		Strong	1,186.036	390	0.001	22.286	6	0.001	0.928	−0.001	0.922	0.001	0.060	0.000	0.0741
		Strict	1,233.315	414	0.001	47.278	24	0.003	0.926	−0.002	0.925	−0.003	0.059	0.001	0.0837
DASS-8	Education	Configural	157.803	68	0.001				0.973		0.967		0.048		0.0659
		Metric	158.825	73	0.001	1.021	5	0.961	0.974	0.001	0.970	−0.003	0.045	0.003	0.0651
		Strong	163.449	79	0.001	4.624	6	0.593	0.975	−0.001	0.973	−0.003	0.043	0.002	0.0686
		Strict	177.112	88	0.001	13.663	9	0.135	0.973	0.001	0.974	−0.001	0.042	0.001	0.0735
DASS-12		Configural	541.567	174	0.001				0.929		0.919		0.061		0.0839
		Metric	545.488	183	0.001	3.921	9	0.917	0.930	−0.001	0.924	−0.005	0.059	0.002	0.0872
		Strong	551.762	189	0.001	6.274	6	0.393	0.930	0.000	0.927	−0.003	0.058	0.001	0.0916
		Strict	570.557	204	0.001	18.795	15	0.223	0.929	0.0001	0.931	−0.004	0.056	0.002	0.0976
DASS-21		Configural	1,570.145	597	0.001				0.914		0.909		0.054		0.0792
		Metric	1,579.413	615	0.001	9.268	18	0.953	0.914	0.000	0.912	−0.003	0.053	0.001	0.0812
		Strong	1,587.843	621	0.001	8.430	6	0.208	0.914	0.000	0.913	−0.001	0.052	0.001	0.0828
		Strict	1,631.604	645	0.001	43.760	24	0.008	0.912	0.002	0.914	−0.001	0.052	0.00	0.0904
DASS-8	Employment	Configural	90.001	32	0.001				0.982		0.968		0.056		0.203
		Metric	94.891	37	0.001	4.890	5	0.429	0.982	0.000	0.973	−0.005	0.052	0.004	0.213
		Strong	103.106	43	0.001	8.215	6	0.223	0.981	0.001	0.975	−0.002	0.050	0.002	0.245
		Strict	122.100	52	0.001	18.994	9	0.025	0.978	0.003	0.976	−0.001	0.049	0.001	0.292
DASS-12		Configural	404.982	96	0.001				0.938		0.915		0.075		0.0476
		Metric	413.900	105	0.001	8.918	9	0.445	0.938	0.000	0.922	−0.007	0.072	0.003	0.0500
		Strong	428.248	111	0.001	14.348	6	0.026	0.936	0.002	0.924	−0.002	0.071	0.001	0.0535
		Strict	448.730	126	0.001	20.482	15	0.154	0.935	0.001	0.932	−0.008	0.067	0.004	0.0561
DASS-21		Configural	1,133.240	366	0.001				0.929		0.918		0.061		0.0410
		Metric	1,160.683	384	0.001	27.443	18	0.071	0.928	0.001	0.921	−0.003	0.060	0.001	0.0438
		Strong	1,175.832	390	0.001	15.194	6	0.019	0.927	0.001	0.921	0.000	0.060	0.000	0.0457
		Strict	1,219.078	414	0.001	43.246	24	0.009	0.925	0.002	0.924	−0.003	0.058	0.002	0.0487
DASS-8	Country	Configural	91.407	32	0.001				0.978		0.961		0.057		0.0232
		Metric	92.290	37	0.001	0.883	5	0.971	0.979	−0.001	0.969	−0.008	0.051	0.006	0.0233
		Strong	213.162	43	0.001	120.871	6	0.001	0.936	**0.043**	0.917	**0.052**	0.083	**−0.032**	0.0501
		Strict	252.328	52	0.001	39.166	9	0.001	0.925	0.011	0.919	−0.002	0.082	0.001	0.0522
DASS-12		Configural	431.680	96	0.001				0.922		0.892		0.078		0.0529
		Metric	441.663	105	0.001	9.983	9	0.352	0.921	0.001	0.901	0.001	0.075	0.003	0.0539
		Strong	577.042	111	0.001	135.379	6	0.001	0.891	**0.030**	0.871	**0.030**	0.086	−0.011	0.0711
		Strict	643.813	126	0.001	66.771	15	0.001	0.879	0.012	0.873	−0.002	0.085	0.001	0.0719
DASS-21		Configural	1,244.101	366	0.001				0.904		0.890		0.065		0.0467
		Metric	1,263.384	384	0.001	19.283	18	0.375	0.904	0.000	0.895	−0.005	0.063	0.002	0.0471
		Strong	1,430.786	390	0.001	167.402	6	0.001	0.887	0.017	0.878	0.017	0.068	−0.005	0.0668
		Strict	1,528.048	414	0.001	97.263	24	0.001	0.879	0.008	0.877	0.001	0.069	−0.001	0.0687
DASS-8	Relationship (spouse/child)	Configural	98.766	32	0.001				0.978		0.961		0.063		0.0222
		Metric	103.065	37	0.001	4.299	5	0.507	0.978	0.000	0.967	−0.006	0.058	0.005	0.0221
		Strong	115.920	43	0.001	12.855	6	0.045	0.976	0.002	0.968	−0.001	0.057	0.001	0.0288
		Strict	134.804	52	0.001	18.885	9	0.026	0.972	0.004	0.970	−0.001	0.055	0.002	0.0365
DASS-12		Configural	375.451	96	0.001				0.941		0.919		0.074		0.0438
		Metric	386.799	105	0.001	11.348	9	0.253	0.940	0.001	0.925	−0.006	0.071	0.003	0.0438
		Strong	410.290	111	0.001	23.491	6	0.001	0.937	0.003	0.925	0.000	0.072	−0.001	0.0465
		Strict	429.829	126	0.001	19.539	15	0.190	0.936	0.001	0.933	−0.008	0.068	0.004	0.0465
DASS-21		Configural	1,116.091	366	0.001				0.926		0.915		0.062		0.0363
		Metric	1,141.408	384	0.001	25.318	18	0.116	0.926	0.000	0.919	−0.004	0.061	0.001	0.0360
		Strong	1,157.691	390	0.001	16.283	6	0.012	0.925	0.001	0.919	0.000	0.061	0.000	0.0392
		Strict	1,200.414	414	0.001	42.723	24	0.011	0.923	0.002	0.922	−0.003	0.060	0.001	0.0428
DASS-8	Type of Dementia	Configural	73.426	32	0.001				0.988		0.978		0.048		0.0194
		Metric	75.085	37	0.001	1.659	5	0.894	0.989	−0.001	0.983	−0.005	0.043	0.005	0.0195
		Strong	81.576	43	0.001	6.491	6	0.370	0.989	0.000	0.985	−0.002	0.040	0.003	0.0254
		Strict	111.745	52	0.001	30.169	9	0.001	0.982	0.007	0.981	0.004	0.045	−0.005	0.0402
DASS-12		Configural	404.163	96	0.001				0.941		0.920		0.075		0.0481
		Metric	407.589	105	0.001	3.427	9	0.945	0.943	−0.002	0.928	−0.008	0.071	0.004	0.0481
		Strong	410.935	111	0.001	3.345	6	0.764	0.943	0.000	0.932	−0.004	0.069	0.002	0.0560
		Strict	448.012	126	0.001	37.077	15	0.001	0.939	0.004	0.936	−0.004	0.067	0.002	0.0560
DASS-21		Configural	1,114.320	366	0.001				0.934		0.924		0.060		0.0367
		Metric	1,126.020	384	0.001	11.700	18	0.862	0.935	−0.001	0.928	−0.004	0.058	0.002	0.0372
		Strong	1,130.640	390	0.001	4.620	6	0.593	0.935	0.000	0.930	−0.002	0.058	0.000	0.0406
		Strict	1,197.276	414	0.001	66.636	24	0.001	0.931	0.004	0.930	0.000	0.058	0.000	0.0507
DASS-8	Level of dependency	Configural	85.979	32	0.001				0.984		0.971		0.054		0.0164
		Metric	94.700	37	0.001	8.721	5	0.827	0.982	0.002	0.973	−0.002	0.052	0.002	0.0170
		Strong	99.625	43	0.001	4.925	6	0.554	0.983	−0.001	0.978	−0.005	0.048	0.004	0.0174
		Strict	106.294	52	0.001	6.669	9	0.671	0.984	−0.001	0.982	−0.004	0.043	0.005	0.0179
DASS-12		Configural	375.451	96	0.001				0.941		0.919		0.074		0.0421
		Metric	386.799	105	0.001	11.348	9	0.253	0.940	0.001	0.925	−0.006	0.071	0.003	0.0412
		Strong	410.290	111	0.001	23.491	6	0.001	0.937	0.003	0.925	0.000	0.072	−0.001	0.0438
		Strict	429.829	126	0.001	19.539	15	0.190	0.936	0.001	0.933	−0.008	0.068	0.004	0.0465
DASS-21		Configural	1,119.346	366	0.001				0.932		0.922		0.060		0.0479
		Metric	1,137.433	384	0.001	18.087	18	0.450	0.932	0.000	0.926	−0.004	0.059	0.001	0.0508
		Strong	1,153.591	390	0.001	16.157	6	0.013	0.931	0.001	0.926	0.000	0.059	0.000	0.0537
		Strict	1,179.034	414	0.001	25.443	24	0.382	0.931	0.000	0.930	−0.004	0.057	0.002	0.0587
DASS-8	Receiving	Configural	71.359	32	0.001				0.988		0.979		0.046		0.0180
	help	Metric	74.438	37	0.001	3.080	5	0.688	0.988	0.000	0.982	−0.003	0.042	0.004	0.0193
		Strong	77.536	43	0.001	3.097	6	0.797	0.989	−0.001	0.986	−0.004	0.038	0.004	0.0231
		Strict	80.204	52	0.001	2.669	9	0.976	0.991	−0.002	0.991	−0.005	0.031	0.007	0.0234
DASS-12		Configural	414.540	96	0.001				0.937		0.914		0.076		0.0398
		Metric	418.887	105	0.001	4.346	9	0.887	0.938	−0.001	0.922	−0.008	0.072	0.004	0.0399
		Strong	438.250	111	0.001	19.363	6	0.004	0.936	0.002	0.923	−0.001	0.072	0.000	0.0441
		Strict	455.058	126	0.001	16.809	15	0.330	0.935	0.001	0.932	−0.009	0.068	0.004	0.0460
DASS-21		Configural	1,166.435	366	0.001				0.927		0.916		0.062		0.0402
		Metric	1,182.852	384	0.001	16.418	18	0.563	0.927	0.000	0.920	−0.004	0.060	0.002	0.0420
		Strong	1,194.925	390	0.001	12.073	6	0.060	0.927	0.000	0.921	−0.001	0.060	0.00	0.0428
		Strict	1,227.466	414	0.001	32.541	24	0.114	0.926	0.001	0.925	−0.004	0.059	0.001	0.0449

χ ^2^, chi-square; df, degrees of freedom; CFI, comparative fit index; TLI, Tucker–Lewis index; RMSEA, root mean square error of approximation; CI, confidence interval; SRMR, standardized root mean residual, values in bold indicate variance or tendency toward variance.

### Results of known-group validity and discriminant validity tests

Table 3 indicates significantly higher scores of all the DASS scales and their subscales among female respondents and those caring for dependent patients as hypothesized. Contrary to expectations, distress levels did not significantly vary according to the type of dementia. Also, respondents receiving help demonstrated higher scores of the DASS-8/DASS-12/DASS-21 than those who did not receive help (all *p*-values < 0.001). Adult children caregivers expressed significantly higher levels of distress than spouse caregivers.

**Table 3:** Known-group validity of the Depression Anxiety Stress Scales (DASS-8, DASS-12, and DASS-21) among dementia family caregivers.

**DASS versions**	**Gender**		**Dependency level**		**Receiving help**		**Relationship**		**Dementia type**	
	***U***	***z***	***U***	***z***	***U***	***Z***	***U***	***z***	***U***	***z***
DASS-8	17,268.0***	−4.59	20,755.0***	−3.56	29,328.0**	−5.26	19,325.5***	−3.33	39,309.5	−0.50
Depression	19,232.0***	−3.32	22,206.5**	−2.66	30,074.0**	−4.90	19,585.5***	−3.18	40,106.5	−0.09
Anxiety	16,333.5***	−5.23	20,627.5***	−3.66	29,215.0**	−5.35	19,398.5***	−3.30	38,408.0	−0.98
Stress	19,165.0**	−3.39	21,642.5**	−3.04	32,017.5**	−3.93	21,351.5*	−1.98	38,719.5	−0.80
DASS-12	16,452.5***	−5.13	20,835.0***	−3.50	28,614.0**	−5.63	18,907.5***	−3.62	38,779.0	−0.77
Depression	17,260.5***	−4.61	21,783.0**	−2.92	29,357.5**	−5.26	19,429.0**	−3.27	38,762.5	−0.78
Anxiety	15,847.5***	−5.54	20,514.0***	−3.72	28,690.5**	−5.60	18,618.0***	−3.83	38,647.0	−0.84
Stress	19,122.5**	−3.38	21,675.0**	−2.99	30,515.0**	−4.67	20,497.5*	−2.54	39,258.0	−0.53
DASS-21	16495.0***	−5.10	20598.0***	−3.65	28696.0**	−5.58	18995.5***	−3.56	39482.5	−0.41
Depression	17,611.0***	−4.37	21,609.5**	−3.02	29,316.0**	−5.27	19,700.5**	−3.08	39,211.5	−0.55
Anxiety	15,868.5***	−5.52	20,352.0***	−3.81	28,649.5**	−5.61	18,726.5***	−3.75	38,741.0	−0.79
Stress	17,731.0***	−4.29	21,271.0**	−3.23	29,717.0**	−5.06	19,955.0**	−2.91	39,300.0	−0.51

DASS, Depression Anxiety Stress Scale; U, Mann–Whitney U-test.

^*^, ^**^, ^***^Differences are significant at a level of 0.05, 0.01, and 0.001, respectively.

Based on the lenient limit of the HTMT ratio of correlations (< 0.90), the depression and anxiety subscales of the DASS-8 and the DASS-21 were distinct from each other (HTMT ratio = 0.89 and 0.90, respectively). Meanwhile, the depression and anxiety subscales of both measures expressed an overlap with the stress subscale. As for the DASS-12, all its subscales had perfect correlations with one another (Supplementary materials), except for the anxiety and stress subscales, which were marginally distinct from each other (HTMT ratio = 0.88).

### Results of tests of reliability, convergent validity, and criterion validity

Table 4 shows adequate reliability of the DASS-8/DASS-12/DASS-21 (coefficient alpha = 0.93, 0.95, and 0.97, respectively) and their subscales (coefficient alpha ranging from 0.77 to 0.95). For the three scales, item–total correlations were considerably high, with no increase in reliability up on item deletion from any measure. The shortened versions and their subscales strongly correlated with the parent scale/subscales, suggesting adequate convergent validity. As expected, all the DASS measures had strong positive correlations with the UCLALS3, which supports their criterion validity.

**Table 4:** Internal consistency, convergent validity, and criterion validity of the Depression Anxiety Stress Scale (DASS) 21, DASS-12, DASS-8, and their subscales among dementia family caregivers.

	**DASS-8**	**Depression**	**Anxiety**	**Stress**	**DASS-12**	**Depression**	**Anxiety**	**Stress**	**DASS-21**	**Depression**	**Anxiety**	**Stress**
MD (IQR)	15 (8–19)	6 (3–8)	4 (1–7)	4 (2–5)	21 (12–28)	7 (4–10)	6 (2–9)	8 (6–10)	38 (19–49)	13 (7–17)	10 (4–16)	14 (9–17)
Coefficient alpha	0.933	0.850	0.904	0.773	0.948	0.866	0.902	0.859	0.973	0.929	0.945	0.923
Range of corrected item–total correlations	0.739–0.823	0.699–0.743	0.802–0.822	0.633	0.717–0.801	0.701–0.756	0.752–0.816	0.635–0.738	0.701 −0.850	0.718–0.806	0.742–0.858	0.730–0.788
Range of alpha if–item–deleted	0.920–0.925	0.770–0.818	0.853–0.870	—-	0.942–0.946	0.812–0.839	0.861–0.884	0.806–0.854	0.971–0.972	0.914–0.923	0.933–0.943	0.907–0.914
Correlation with the corresponding subscale of the DASS-21	–	0.945**	0.948**	0.928**	–	0.974**	0.968**	0.967**	–	–	–	–
Correlation with the DASS-21	0.977**	0.894**	0.929**	0.852**	0.987**	0.945**	0.903**	0.903**	–	0.959**	0.954**	0.943**
Correlation with UCLALS3	0.737**	0.688**	0.724**	0.594**	0.735**	0.729**	0.673**	0.644**	0.748**	0.731**	0.724**	0.679**

MD, median; IQR, interquartile range; DASS, Depression Anxiety Stress Scale; UCLALS3, the 3-item University of California, Los Angeles, Loneliness Scale-version 3.

^**^Correlations are significant at a level of 0.01.

## Discussion

This study examined the psychometric properties of three DASS measures among dementia family caregivers, with the aim of providing a credible short version that may be promptly used for detecting mental distress in this vulnerable population. Compared with the DASS-12 and the DASS-21, the three-factor structure of the DASS-8 had the best fit. It also expressed adequate measurement equivalence, reliability, convergent validity, discriminant validity, and criterion validity relative to the longer versions.

As shown in Table 2, all the DASS measures were invariant at all levels across a wide range of participant characteristics (gender, education, employment, relationship with care recipient, type of dementia, level of dependency, and receiving help with care giving). The shortened versions of the DASS were or tended to be non-invariant at the scalar level across the country of residence. Non-invariance of these measures has been previously reported across English-speaking and Ghanian individuals. Nonetheless, they were invariant across English-speaking respondents from Australia and the United States (38). Likewise, the DASS-21 was non-invariant across countries with different languages, locations, economy, and cultural backgrounds (e.g., Poland and Russia vs. the United States and the United Kingdom as well as Germany vs. Pakistan) (28, 29). In the current study however, the respondents were recruited from a limited border area where people from both countries could fluently speak Italian. Thus, it is not expected that participants in this sample present major cultural variations. Therefore, non-invariance of the shortened version across country in the present study may be partially attributed to the considerably small number of participants in the Swiss group relative to the Italian group. Variations in group and sample sizes are reported to wrongly affect scale score equivalence. Many typical fit criteria may not be suitable in such contexts (49, 50). Moreover, the number of items, degree of factor over determination, and the level of indicator communalities can considerably affect measure fit and scale invariance (49). In this respect, models with small degrees of freedom (df) tend to express inflated RMSEA (51, 52). This was notable in the model examining the DASS-8 compared with that of the DASS-12, which also exhibited inflation in ΔCFI—a more reliable measure of misfit in small scales than RMSEA (51, 52). Accordingly, future studies investigating the invariance of these shortened versions need to take the influence of sample size on scale equivalence into consideration.

As for the tests of known-group validity, the DASS measures significantly identified distressed groups (Table 3). As expected, female carers and those caring for ADL-dependent patients had higher distress levels than male carers and those caring for autonomous patients, with no difference between Alzheimer's disease and other types of dementia. More than half the respondents (58.7%) stated that they received help with caring for patients with dementia. In contradiction to our hypothesis, those receiving help expressed greater levels of distress than those who did not receive help. Dementia caregiving is primarily provided by families (in up to 65% of cases) (18), and the worst levels of caregiver distress are largely reported among those caring for severe cases than those caring for mild cases (18, 19). For those who reported receiving help, 55.3% of their patients had Alzheimer's disease, and 79.7% of patients were not able to perform ADL. Therefore, ADL dependency, which may be associated with dementia severity, is the possible cause of distress in this group. In addition, caregiving is also reported to negatively influence the health of caregivers (14). Accordingly, those who perceive their health as deteriorating as a result of extensive caregiving are more likely to ask for help. For 54.9, 38.8, and 6.3% of the respondents who indicated that their patients received supplementary care, care was provided by another relative, nurse, or friend, respectively. Caregiver distress during the COVID-19 pandemic is associated with fear of the absence of paid caregivers as well as fear that contact with people who assist with instrumental activities may transmit this virulent infection to their patients (18). In addition, caregiving interferes with adult children's work schedule, while hiring health professionals to care for this chronic condition may represent a persistent financial burden (14).

Based on an existing review, we hypothesized that spouse caregivers would express higher levels of distress than adult children caregivers (8). Paradoxically, the latter demonstrated more distress than spouse caregivers. This finding can be related to the fact that the pandemic has created a lot of challenges for younger groups such as increased time spent caring for their children due to school closure, loss of jobs/income, and social isolation imposed by the lockdown. Meanwhile, spouses are older and more likely to be retired, with a greater possibility of being more home-bound than the youth. Moreover, age is reported as a protective factor against distress and trauma during the pandemic (15).

Discriminant validity tests show that the depression and anxiety subscales of the DASS-8 and the DASS-21 were distinct from each other. Thus, the DASS-8/DASS-21 may be used to distinguish the symptoms of depression from those of anxiety, albeit the stress subscale was overlapping with both subscales in both measures. A total of two previous studies revealed that most subscales of the DASS-8 were distinct from each other—the stress and anxiety subscales were overlapping with one another (37, 38). However, that was not true for the DASS-12, which could only discriminate anxiety symptoms from stress symptoms in the current study. All the DASS measures positively correlated with the UCLALS3 at the same level of significance, indicating usefulness of the DASS-8, DASS-12, and DASS-21 as criterion variables. All these measures also demonstrated comparably adequate internal consistency and convergent validity, as noted by high values of item–total correlations and correlations of the shortened versions with the parent scale/subscales.

This study expands the literature by using various techniques to examine three DASS measures in a particularly distressed group (dementia family caregivers) from two European countries during the COVID-19 outbreak. Given that the psychometrics of the DASS-8 were adequately similar to those of longer DASS scales, it may be easier to frequently screen for possible psychopathology among dementia caregivers using this brief version. Scale brevity is a key advantage, especially for a scale that inherits the validity of the parent scale since response rates decrease with the administration of long scales (51, 52). The study also enjoys the merit of repurposing already available public data to generate new knowledge without consuming extensive economic and intellectual resources. Despite these advantages, a number of limitations should be noted. The recruitment and data collection methods entail a risk for selection bias where only those using social media and a smart phone could participate in the present study. Another possibility of selection bias stems from the fact that most of the participants of the study are women. Women may vary in the extent of their emotional experience and expression of distress from men (53)—in fact, greater levels of distress among women were detected in our analysis. Nonetheless, the DASS measures were invariant across genders, indicating that they are less likely to be biased by women's tendency to express more negative emotions than men. Additionally, the UCLALS3 expressed adequate internal consistency, denoting that it enjoys the basic psychometric properties of a scale. Nevertheless, statistics on the different types of validity of this three-item scale as an adequate measure of loneliness are lacking, putting our test of criterion validity at jeopardy. The results may not be generalized because of the cross-sectional design, the convenience sample, and the limited time and location of data collection (during an early stage of a prolonged pandemic and from a border region between Italy and Switzerland). Examining the invariance of the DASS measures across those two border regions may not be sufficient to reflect invariance across countries. Although the sample size meets the requirements for CFA analysis based on 21 items of the DASS-21 (20 responses per one item), multigroup comparisons across countries may not be that robust because of the vivid variation in the number of respondents in the country groups. Using an adequate number of groups and participants in groups is necessary for future investigations to properly examine the measurement invariance of the DASS-8/DASS-12 across more European and non-European countries. Multiple participation may represent a threat to data integrity since the survey was conducted online, and there is no information available on the control of the number of participations per person. Moreover, the respondents were included based on self-reporting their state as family caregivers of people with dementia. Not using credible references (e.g., the medical record for the care-recipients) to confirm that the respondents were really caregivers may entail a risk for selection bias.

## Conclusion

The DASS-8 displayed a better factor structure than longer versions, and all its other psychometrics (measurement invariance, reliability, convergent validity, criterion validity, and known-group and discriminant validity) were adequate, compared with longer versions. Because the course of dementia is chronic and progressive, considerable attention should be paid to the identification of high levels of distress among caregivers, especially female carers, adult children of patients with dementia, those with highly dependent patients, and those who ask for supplementary care. The DASS-8 can be a useful brief measure for achieving this aim.

## Data availability statement

Publicly available datasets were analyzed in this study. The dataset supporting the conclusions of this article is available in Zenodo repository at https://zenodo.org/record/4748652#.YdbwiWhBw2w.

## Ethics statement

Ethical review and approval was not required for the study on human participants in accordance with the local legislation and institutional requirements. The participants provided their written informed consent to participate in this study.

## Author contributions

All authors listed have made a substantial, direct, and intellectual contribution to the work and approved it for publication.

## Conflict of interest

The authors declare that the research was conducted in the absence of any commercial or financial relationships that could be construed as a potential conflict of interest.

## Publisher's note

All claims expressed in this article are solely those of the authors and do not necessarily represent those of their affiliated organizations, or those of the publisher, the editors and the reviewers. Any product that may be evaluated in this article, or claim that may be made by its manufacturer, is not guaranteed or endorsed by the publisher.
